# Extra-Limites

**Published:** 1841-01-01

**Authors:** 


### EXTRA-LIMITES.

### Proceedings of the Phrenological Society at Glasgow, in September last.

We perceive that this Section has been very active and successful at Glasgow, during the meeting of the British Association. We select a couple of specimens from their proceedings.

" *Monday, September* 21, 1840."

" The Hall was crowded with ladies and gentlemen.

Mr. Atkinson read a communication from Mr. R. Cull, of London, detailing a case of precocious musical talent, in the history of the *Infant Sappho,* Louisa Vinning. She was born at Kingsbridge, Devonshire, in November, 1836, being now (Sept. 1840) three years and ten months old. Her father, John Vinning, is a good musician : he sings, and plays well on the piano forte and violin, and, having also exhibited his musical talent at a very early period, he was educated for a musician, at the expense of Mr. Garrow. Mr. Vinning has two brothers, of considerable musical talent, who have left their business to make music their occupation. One is a violinist, the other an organist. Mr. Vinning's father possesses a natural talent for music, which he manifested by playing the flute, in the band of a volunteer regiment, for several years. He knows nothing of the technical language of music—he played entirely by ear, and he kept tune and time well.

Louisa Vinning, surnamed by Mr. Parry the Infant Sappho, enjoyed music at a very early age. ' She was only nine months old,' her father states, ' when I first observed the intense delight she derived from music: when crying, the sounds of a musical instrument immediately soothed her, her whole frame moving in unison with the measure, and her face beaming with enjoyment. I played to her occasionally on the violin. I took the opinion of several medical men on the propriety of indulging her in this kind of amusement, lest she should be injured by too early excitement. Their advice was, to give her gentle exercise in singing, and to guard against late hours.' She sang before she could speak. Her passion for music increased, until she seemed to require an atmosphere of music to exist.

In the early part of 1839 she was discovered to have walked in her sleep, and, to prevent accidents, she was afterwards put to sleep on a sofa in the sitting-room until the family retired to rest; she frequently sang in her sleep, and, one evening, when only two years and eight months old, she sang, sweetly and distinctly, a melody perfectly new to her father, and repeated it several times, so that he wrote it down, gave it to Mr. Blockley, who arranged it, wrote the poetry, symphonies, and accompaniments, and called it the *Infant's Dream.* Mr. Thalberg, the celebrated musician, in a letter dated 11th December, 1839, speaks of her astonishingly correct singing, and her pleasing voice. Sir George Smart, in a letter dated 3d April, says, ' I beg leave to state that I consider her a most wonderful child, possessing strong feeling for music, with an extraordinary correct ear both for time and tune; her singing is perfectly natural, without effort, and her infantine manners and childish appearance prove her extreme youth.' Mr. Moschelles says, in a letter dated 29th March, 1840, ' she appears to me, not only to be most liberally gifted with a voice of unusual compass, but also with a sensitiveness of organisation, whether as concerns the power of correctly retaining melodies, or of reproducing intervals, very remarkable, being only three years and a half old.'

She sung before the Queen and Court at Buckingham Palace, on 3d August, 1840, and received substantial proofs of the Queen's delight at her talent. She is now singing three nights a-week in the Lecture Theatre of the Polytechnic Institution. She sings the musical *sounds* of the melodies without words; and repeats any Italian air, after hearing it only three or four times. Her style of singing is very remarkable for similarity to our first opera singers. It is appropriately supported by the adoption of the natural language, gesture, &c. to express the sentiment of the air she sings. In her graceful though infantine action she is often very expressive; but, like most public singers, there is commonly a redundancy of action, and that, too, of an exaggerated nature. Her public singing at the Polytechnic Institution commonly comprises the following:—

1. An Italian Air.
2. The Infant's Dream.
3. The proof of her power to sing passages struck on the Piano on the instant, which frequently terminates in some Italian air.
4. Her power of changing the style and key of music without the usual preparation, in which she passes at once from some Italian to an English, thence to a Scotch, and finally to an Irish air.
5. An Italian Air.
6. Finale, part of a harmony in the National Anthem of God save the Queen.

All her talent is natural, for hitherto she has received no technical instruction in music. Her voice is two octaves in compass; the lower notes are very sweet in quality, and she possesses great power of voice. She can introduce occasional sharps and flats with great precision and elegance. When false notes were purposely played to try her, she invariably ceased, and evinced some anger.

She is an engaging child, and, from her elegant movements, is much admired. She has a great talent for dancing also. She is very energetic, her general activity is great, her feelings powerful, and very exciteable. She is self-willed, destructive, very ready to talk, and very arch.

The essay then stated the Phrenological measurements of the head, all of which were very large for a child of her age. She is of dark complexion, dark brown eyes, brown hair, slender form, restless movement of body and eyes, and rapidity of action, which denote great cerebral activity. The temperament is *Bilio-nervous*. The basilar region of the brain is large, but the coronal predominates. The lateral is very large at Destructiveness and Secretiveness. The anterior is also large. The profile much resembles the profile portrait of Clara Fisher. In so large a sized head there are no small organs. Those *very large* are *Secretiveness, Destructiveness, Benevolence, Firmness, Love of Applause, Imitation, Melody, Tune, Comparison,* the others are *large*.

This head is interesting musically as an example of the energetic manifestation of musical talent. It is also interesting as it so nearly corresponds, in its present powers, with the infantine powers of Mozart, Crotch, and Kellner, as quoted in the Phrenological Journal, new series. The case is interesting, as pointing towards a circumstance in the production of precocious talent. Mozart, Crotch, Kellner, and this child, are each offspring of musical fathers; and the two latter of musical paternal grandfathers. Other circumstances also operate as causes, for the offspring of all musicians are not musical, and but few are precocious musicians.

After the reading of the case, some interesting remarks were made by Mr. Atkinson, Dr. Gregory, Mr. De Ville, Mr. Simpson, and Mr. Combe, and several other cases of precocious musical talent were alluded to by the different speakers. Dr. Gregory said it was a great pity that this child should be subjected to such increased activity of brain, which, it was well known to phrenologists, was very liable to produce disease, and lead to premature death; and Mr. De Ville stated that he had intimated to the parents of the infant Lyra, another musical child, that the exertion of brain to which she was subjected, in consequence of her public exhibitions, would infallibly bring on premature decay; and, as her parents did not listen to his advice, which was agreeable to the phrenological doctrines, the child, by the continued and severe exercise of her brain, fell into disease and died at an early age.

Mr. Simpson then read a most interesting and highly instructive essay on 'the phrenological explanation of the result of a change of treatment of youth, from animal and violent to moral and benevolent, with illustrative cases.' This was listened to with deep attention, and seemed to make a great impression on the audience. Mr. Simpson began by showing how much phrenology had done in introducing and systematising the only education worthy the name—a character-forming, a humanity-improving education—an education which rightly trains and exercises the faculties which phrenology has distinguished as primitive—each faculty upon the objects in nature related to it. He showed that the faculties in activity in one individual rouse the same faculties, by sympathy, in another; and hence the vital importance that the trainer of youth should manifest only those faculties which it is desirable to strengthen in his pupil, and to restrain and repress those which are never called forth, in abuse, but to injure or annoy. The pupil, therefore, should never see the teacher, nor the child the parent, angry, loud, or violent; never insolent and tyrannical; in phrenological language, manifesting *self-esteem, combativeness,* and *destructiveness:* but, on the contrary, should witness only justice, kindness, with temperate firmness. *Benevolence,* which is moral power, ought to be the great engine of education. It is power with man and beast. The Arab never strikes his horse—yet the beautiful Arabian, which lives, eats, and sleeps with his master, is the best educated horse in the world. Mr. S. then proceeded to show that the treatment of the young has hitherto been the reverse of all this. It has been commanding, tyrannical, and violent. He drew a picture of the flogging and fagging system, and the cowardly frauds which it engendered in school, and the coarse and brutal, and especially puerile, character it produced in society; witness the police reports of our *adult* school-boys. Some boys either passively or actively resist the violent system, and are pronounced unmanageable. The boy, we may suppose, has been sent from a strict school, as the severe were called, to one still more strict; and he is duly returned from each with an apology, that he defied all authority, and, having arrived at the point of beating and kicking his master, was beyond his management. This unmanageable boy, we shall suppose, is seen by a phrenologist, who discovers an excellent moral and intellectual organization, in connexion with a large animal; and knowing that, while the animal alone has been exercised, the others, especially the moral, have been left in abeyance, he at once declares that the unfortunate boy is a *mismanaged,* not an unmanageable subject. He proposes a complete change of treatment. He addresses himself to the higher sentiments and intellect, no longer excites the low and violent feelings, and soon produces a complete change of conduct. This is not a mere theory, for many examples might be adduced of its practical value. Mr. S. mentioned a gentleman of the most active generosity and beneficence, who at school was mistaken, by those who could not read the better faculties he possessed, for an incarnation of the evil one. He was beaten at school, but always beat again, and was repeatedly sent home as a hopelessly unmanageable boy. Subjected to the old system of '*taming,*' he was found as untameable as the Hyena. Left to himself, his higher feelings began to work spontaneously from their own internal energy; and now they take the lead so perfectly that the animal faculties, which formerly beat his teachers, merely supply energy in the prosecution of his philanthropic views. He is himself a well-informed phrenologist, and knows the process of his own transformation.

Mr. S. then proceeded to describe two most gratifying experiments made under his own roof—first, upon two young ladies of high rank, at the request of the parents, placed in his family, and educated along with two of his daughters, who were of the same age. The whole process was kindness, confidence, and intellectual guidance, by which a large amount of waywardness and selfishness gradually gave way to the growth of excellent moral sentiments, till the characters were almost metamorphosed. The other instance was that of a youth, now past 16, who, a year ago, presented one of the most unmitigated specimens of all the unendurable faults induced by subjection to the violent method of common schools, and the examples of coarseness and brutality there presented. No school could keep him—no home could endure him—yet this youth is born to a large fortune, and a high rank in society. Mr. S. who is intimate with his parents, invited him to his house a year ago, and giving him line, as the anglers say, for a week, became aware of the domestic nuisance the poor boy thought it manly and *funny* to make himself. In one week, he became so intolerable to the family that there was an urgent and general request to send him home again. Instead of this, a trial of better conduct was deliberately proposed to him, and his powers of succeeding explained. His faculties for good, and the miserable error of his previous education, were all detailed to him. He was enlisted as a principal agent in his own reform, and the change was the doing of a day—so that, for three weeks more that he remained, his manners became quiet, obliging, and gentlemanly. Kind and confiding treatment had great power over him; and treated, on his return home, in the same way, the sudden change surprised his friends as much as if it had been wrought by miraculous agency. It may be believed that earnest application was made for his return to the scene of an experiment so auspiciously begun, if it could be made to suit Mr. S.'s family arrangements. The experiment was too interesting to the experimenter to be declined; and the youth has now been the intimate of Mr. S.'s family for a year; and, subjected to its influences, treated as a gentleman, and allowed all proper liberty and indulgence, he gives ready obedience to the authority of good sense and good feeling; and no more dreams of rudeness, or any form of old school misconduct, than he did of common civility in his former state. The moral command of kindness over him is manifold greater than was ever yet achieved by severity. There is much yet to do with feelings and habits so perverted as his were; but he is already a *comfortable inmate*, and within the range of yet higher moral appliances. Mr. S. promised to report further progress to the Association when it meets again, and stated that he possesses full liberty from the parents to make any use of the case he may think proper, for educational good.

Mr. S. concluded his eloquent and most instructive essay, with stating that he offered this and the other cases, to show that the educational system—based, he was proud to say, on phrenological principles—of which he was a humble disciple and unworthy advocate, is *practical*, and not the mere chimera which its uninformed, prejudiced, or interested enemies represent it. He was called a visionary—he begged to be judged by his practice, not merely in these cases, but in numerous seminaries now founded on his principles. He retorted the title of visionaries on his opponents, who persevered in a system which has produced nothing but evil, and has retarded, and is retarding, the progress of society. Mr. S. warned his hearers that, at the age of 15, the softening and particular influences of a private family, with a subject or two, or a very few, is necessary. No large school can produce much effect on a boy who has had no *infant training*, and all the *mistraining* of ordinary schools. Mr. S.'s subject of experiment was not in the least improved by being placed at a large school, which had even adopted the most advanced views, from which accordingly his parents were requested to remove him.

Case of apparent Exception to the Rules of Auscultation of Valvular Disease of the Heart, with Explanations of the Anomalies.

Sarah Fright, æt. 28, a pauper residing in the workhouse of the Thanet Union, had laboured for some years under general debility and local disease of several parts. Her greatest suffering, perhaps, was referred to the uterus and neighbouring parts, and she required the habitual use of the catheter. She complained of almost constant palpitation, with pain in the region of the heart, dyspnœa, occasional though unfrequent cough, and the usual train of thoracic symptoms. Frequent and severe headache; nervous system generally deranged.

About two years ago, she was admitted as an in-patient of St. George's Hospital, where she was for the first time subjected to a competent physical examination. Having been sent up to Dr. Hope, he found a diastole murmur over the aortic valves, with a jerking, though very feeble, pulse; and he therefore gave a diagnosis of *aortic regurgitation;* but, as no other murmur was heard, he confined his valvular diagnosis to this circumstance. On other grounds, *dilatation and flabbiness* of the heart were added to the diagnosis. The signs were not observed to undergo any change during a month which she spent in St. George's Hospital.

From that time I have had frequent opportunities of examining her, and having invariably found the aortic regurgitant murmur to be the only one present, I did not anticipate material disease of any other set of valves. She died October 6th, 1840, after the gradual supervention of dropsy and the other ordinary consequences of cardiac disease. It may be added that the pulse became small, weak, intermittent, irregular and unequal—conditions especially connected with disease of the mitral valve, and also with softening of the heart.

*Autopsy.*—The uterus was extensively diseased. There were two quarts of fluid in the pleura. *Pericardium* was healthy and without effusion. *Aorta* was natural. The whole *heart* was dilated, soft, and flabby. *Auricles.* Both were dilated and attenuated, the left to a very considerable degree, and their tissue was softened. *Ventricles.* Both were dilated without thickening, and their tissue was soft, pale, and flabby. *Aortic Valves.* Their expansions were thickened and contracted, and their angles adhered to each other over an extent of about two lines, by which, together with contraction of their depth, they were depressed a quarter of an inch below the level of their angular insertions, and the circumference of the aperture was so far diminished as to admit the thumb only. *Pulmonic Valves,* natural. *Mitral Valve.* Its layers, as well as the chordæ tendineæ, were exceedingly thickened by fibrous tissue, and its aperture contracted, so as to admit the passage of one finger only. Its margin was flat and flexible,—not thick and stiff like a ring,—and its shape was an irregular slit. The anterior lamina was thickened, though not shortened, chordæ overlay the orifice so completely and accurately as effectually to prevent regurgitation,—which was, indeed, further proved by the absence of murmur. *Tricuspid Valve* was equally and similarly contracted, but rather less thickened. The dissection was made by me in the presence of Dr. Hope.

*Remarks.*—This case presents a greater number of exceptions to general rules of valvular auscultation than I have seen, or found recorded, in any other single instance. Dr. Hope has, in his work, pointed out, in different cases, all the exceptions alluded to; but neither he nor any other writer, to the best of my knowledge, has met with so considerable contraction of the aortic orifice *constantly* unattended with murmur.

I shall notice these several exceptions in succession, commencing with those of the auricular valves.

Both these valves, in the case before us, were so contracted as to admit the passage of one finger only; yet they were not productive of diastolic murmur, (*i. e.* with the second sound, near the apex of the heart.) The same was noticed by Dr. Hope in the parallel case of Christian Anderson; also in those of George Sharpe, Mrs. C——n, and Elizabeth Dennis, in which the mitral valve alone was materially diseased; and he states that, " ever since he has been able to detect aortic regurgitation with certainty, he has found the mitral diastolic murmur to be exceedingly rare," having hitherto met with it in two cases only, viz. N——, Esq. and John Goff, a case with which I supplied him. He thus converts the previously received general rule of Laennec into the exception, the general rule in his opinion being, that *contraction of the auricular valves is not usually productive of diastolic murmur.* He offers the following explanation of the fact. " Much investigation has led me to ascribe its feebleness (viz. of the diastolic murmur), when it does exist, and its absence in circumstances where it might have been expected, to the weakness of the current of blood flowing during the diastole from the auricle into the ventricle. This weakness allows the blood to pass in silence through the aperture when only slightly contracted; and, when the weakness is preternaturally augmented by debility of the heart, even a high degree of contraction is not productive of sound."—(*Treatise on Dis. of Heart*, 3*d Edit. p.* 79.) This explanation is perhaps the only one that can be assigned in the present state of our knowledge, and it is probably the real one. The present case certainly exhibited the circumstances requisite for its application, the patient's circulation being exceedingly weak.

So much for the *diastolic* murmur of the auricular valves: I next proceed to the *systolic* murmur of the same, which was absent in the case before us.

In my account of the dissection I have expressed my belief that the adaptations of the parts of the diseased auricular valves were so accurate and complete as effectually to prevent regurgitation, the margins of the orifice being flat, and the anterior lamina so overlaying the posterior as, by oblique pressure (for these are undoubtedly oblique valves), perfectly to close the aperture. The possibility of this arrangement of parts is so manifest, that it needs no discussion; and I may remark that nothing is more common than moderate fibrous hypertrophy of the mitral valve in cases of hypertrophy of the left ventricle and otherwise, in which we are accustomed to admit, and with propriety, that the valve is efficient. If, then, the valve close, though with a higher degree of disease, it must still be considered efficient as a *closing* valve. It appears to me probable that, in Dr. Hope's case of Mrs. C——n, in whom there was no regurgitant mitral murmur, the valve closed completely, in consequence of the aperture, which only admitted a quill, being in the form of a slit.

But independent of such cases as these, in which it is probable that the valves are capable of closing, there are others, according to Dr. Hope, in which they may be incapable of closure, yet, from mere feebleness of the regurgitant current, not produce a murmur. He says, " When that force (viz. the great force of the ventricular contraction) is much diminished, by softening or by dilatation with attenuation, the murmur may be much more feeble—nay, *sometimes even extinct.* I have, for instance, met with several cases, in which a murmur attended every strong contraction of the ventricle, while the two or three following contractions, so feeble as barely to occasion a pulse, were productive of a valvular click only, without murmur."—(*Treatise, &c.* 3*d edit. p.* 77.) But though such cases prove conclusively that mere feebleness of the regurgitant current may cause the murmur to fail, they would cause no obscurity in diagnosis, because nothing less than an almost complete *intermission of the pulse* suffices to weaken the regurgitant current to the degree required: for we see, by Dr. Hope's cases of Christian Anderson and George Sharpe, to which I could add many of my own, that the highest degrees of dilatation and of softening are insufficient to suspend the murmur during *full* ventricular contractions. In all cases, therefore, the murmur would probably accompany every contraction fully developing the pulse, and this would be sufficient for all purposes of diagnosis.

We may, therefore, dismiss the exceptions to the existence of regurgitant murmurs when the auricular valves are diseased, by the conclusions—1. That when a valve, however diseased, closes completely, it is efficient so far as its closing function is concerned; any evils resulting from simultaneous contraction being a separate question. 2. That where the valve does not close, there will always be a murmur with full contractions, and this will suffice for diagnosis.

I now proceed to the aortic valves. I am not aware of any well-attested case on record, except the one before us, in which there was considerable contraction of the aortic orifice by its valves, without a systolic murmur. Dr. Hope, at page 576 of his work, has referred to the only case within my knowledge that approximates to it. "This (the high key of aortic murmur)," says he, "is well exemplified in another individual at present under my notice, affected with disease of both valves, (aortic and mitral,) in whom there are from two to five beats of the heart accompanied with grating murmur, (mitral,) but no pulse in the radials: then succeeds a stronger shock with a pulse, and a hissing opposite to the aortic valves." Though a post-mortem examination is not attached to this case, there can be no doubt that the hissing murmur was referable to a contraction of the aortic valves or orifice. We have, therefore, such a contraction not producing a murmur whenever the ventricular diastole was feeble; just as we have seen the same to occur, under the last head, in the case of the mitral valve. It accordingly becomes a question whether feebleness of the circulation was sufficient to account for the *constant* absence of murmur in the case before us of Sarah Fright, in whom the aortic valves only admitted the passage of a thumb. In favour of the affirmative, it may be stated that not only was the whole organ dilated, soft and flabby; but that both the mitral and tricuspid valves were so contracted as to admit the passage of one finger only, whence the supply of blood to the ventricles might have been scanty. It is conceivable that this scanty supply, feebly projected by the softened left ventricle, and still further retarded by the constant retrograde pressure of the regurgitant stream, might have passed in silence through an orifice, which, though contracted, was still considerably larger than the mitral aperture, by which the blood was admitted. It is also possible that the infundibuliform shape of the aortic valves contributed to the silence of the passage.

Whether this be thought an adequate explanation or otherwise, it may be stated, independent of any theoretical considerations, that, where the concurrence of so many and so important circumstances as existed in this probably unique case, is requisite to constitute an exception to the general rule, that contractions of the arterial orifices generate a systolic murmur, the exception must necessarily corroborate the rule. To believe otherwise would require a singular share of credulity; nor would less be requisite to imagine that such exceptions would be frequent; or that, when they did occur, they would materially deteriorate diagnosis: for that would generally happen which happened in this case, viz. the associated lesions with their signs would abundantly reveal the leading features of the malady. In the case of Sarah Fright, a stethoscopic examination at once pointed to structural disease of the heart, and to disease that was incurable; faithfully indicated the existence, and one of the seats—certainly the most important—of valvular disease; unveiled the dilatation and softening of the muscular substance; rendered the treatment definite, consistent, and rational; and led to a prognosis which the great instructor, time, has only too accurately verified.

On the other hand, as the notion of infallibility is injurious to cautious research, an exception, however rare, is a salutary check on overweaning confidence.

JAS. FREEMAN.

*Minster, Isle of Thanet, Nov.* 5, 1840.

### ON DISLOCATION OF THE ANKLE JOINT FORWARDS AND BACKWARDS; AND ON REPRODUCTION OF BONE AFTER THE OPERATION OF TREPAN. By *James Douglas*, A.M. Lecturer on Anatomy, College Street, Glasgow. (Read before the Medical Section of the British Association at Glasgow, 19th September, 1840.)

IT is stated by Professor Samuel Cooper, in the last edition of his invaluable Dictionary, that dislocations of the tibia at the ankle forwards and backwards, are not common; and that during fifteen years Dupuytren scarcely met with two or three cases, though he saw some hundreds of lateral dislocations. I have happened, in my short experience, to see three cases of these accidents, the histories of which I now propose to read to the medical section, shewing at the same time the preparations which were made from two of them.

Mr. Adams, in the Cyclopædia of Anatomy, gives it as his opinion, that no satisfactory example is recorded of luxation of the ankle-joint, without fracture of one or both malleoli. He also doubts the possibility of a *complete* luxation of the tibia forwards, so that the inferior articular surface of that bone shall be entirely in front of the articular surface of the astragalus. He conceives that the more common form of this luxation, or perhaps the only one, is what has generally been called a *partial* luxation forwards; when the tibia does not entirely leave the astragalus. Even this form, as I have already remarked, is so rare, that I have ventured to think that any instance of it may be acceptable to the surgical portion of the Association.

A woman, aged 60, died of cancer of the breast in the Glasgow Royal Infirmary, in the year 1834. She had an unreduced dislocation of left ankle-joint forwards, of two years' standing, which I removed and prepared. The cast which I lay before you was taken from the limb after death. It shews the ankle in a state of complete extension, the toes being pointed down. A deep curve is seen behind, where the tendo-Achillis should be straight; the heel is lengthened, and the fore part of the foot is shortened. The anterior edge of the lower end of the tibia makes a projection in front, and a notch exists below it, between it and the division of the foot. The outer ankle is found in its proper place; but the inner one is seen to be thrown forward about three quarters of an inch.

The following is the appearance of the parts on dissection. I describe them with the foot resting on the sole.

Ankle is completely extended, and quite stiff. Tibia rests about three quarters of an inch further forward than it ought. Articulating surface of astragalus is not visible in front, but is felt far back, under the arch of tibia. Anterior edge of tibia is exactly over the articulation of the astragalus with the os naviculare, and nearly three-fourths of an inch above it; so that a small part of scaphoid cavity of tibia behind still rests on the pulley of the astragalus. The tendon of the tibialis anticus by this means runs in a straight line to its insertion at the internal cuneiform bone, instead of curving forwards. Behind, the astragalus projects so much, that the flexor longus pollicis does not run in its groove on the tibia at all. The astragalus and os calcis are in this portion relative to each other, and their posterior ligament is entire. Some additional ossification has taken place on back of tibia, close above the astragalus.

Externally, the external malleolus remains perpendicular in its situation, with its three fibulo-tarsal ligaments entire. A hollow runs obliquely upward and backward from its anterior edge, shewing where a fracture had taken place, the superior anterior portion being thrown forward along with the tibia. Some new bone is deposited on the junction. The peroneal tendons preserve their proper relations.

Internally, the deltoid ligament seems to have been ruptured, though its place is now supplied. The tibio-fibular ligament must have been ruptured, though the new deposits of bone prevent its state from being accurately ascertained.

Neither Sir Astley Cooper nor Dupuytren has ever seen a dislocation of the tibia backwards from the astragalus, nor has any instance been recorded by any other individual. In the article in the Cyclopædia of Anatomy already referred to, a case by Mr. Colles of Dublin is noticed, supposed to be one of dislocation backward; but from the account, I suspect it must have been one of fracture close to the ankle-joint, such as I am now going to describe.

Hugh Macnab, æt. 41, was admitted into the Glasgow Royal Infirmary in July, 1834. Three years before he had fallen through the joists of a new house, upwards of 60 feet, and was struck on anterior aspect of left leg, immediately above ankle, by a plank which fell with him. A fracture above the joint took place, and though he was put up in splints for three months, union was never obtained, and a false joint formed.

On examination, lower portion of tibia, with internal malleolus, was felt attached to astragalus, while shaft of tibia was thrown backward. Considerable doubt existed among the gentlemen who examined the case, whether the fibula was fractured or dislocated, Some thought it was a pure case of dislocation of both bones backward. Leg was three-fourths of an inch shorter than right, and foot seemed very long anteriorly, and very short behind. (See cast.) When he walked, the lower end of the shaft of the tibia pressed against the tendo-Achillis, making it project backward, so as to cause considerable pain. This projection is seen in the cast. The fibula is seen projecting in a similar manner on the outer side. He was thus prevented from working as a labourer, and he insisted on having his foot amputated. The following was the state of the parts as seen upon dissection.

Tibia and fibula (see preparation) are both found to be fractured transversely, immediately above ankle-joint, which remains perfectly entire. Each malleolus remains in its natural situation, with the different tendons lying in their proper grooves. A thin arch of the tibia, not a quarter of an inch in thickness, remains over astragalus, and has formed a ligamentous connexion with its articular surface. Fractured surface has become smooth, and covered with a periosteum.

The shafts of the bones pass backward and downward; their extremities are covered with cartilage, and have received new fibrous capsules, derived from the deep fascia of the leg, in front and on each side of the tendo-Achillis. End of tibia does not rest on the os calcis, but passes backward and downward against the tendon.

In corroboration of the opinion expressed above, that there is no such thing as dislocation of the ankle-joint backward, I may relate a case which Dr. Lawrie has had under his care for some time, and which he asked me to go to see, as an excellent illustration of this subject.

Alexander Laird, æt. 41. Two years and three months ago fell down a coal-pit, a depth of about 20 fathoms, and in addition to other injuries, sustained a compound fracture above right ankle-joint. The wound ran along the inner side of the foot, where a fistulous opening still exists, through which eight pieces of bone have been discharged during the last six weeks. The bones of leg were thrown backward, so that the case was taken for one of dislocation backward, and the injury seemed so severe, that amputation was proposed. To this the patient demurred, and the limb was accordingly put up in splints; but from the unruly and dissipated habits of the patient, could not be kept perfectly reduced. The state of the parts now is as follows.

Toes are pointed down, and leg is shortened, so that he wears a cork sole nearly an inch thick at the heel. There is slight mobility of the toes, but none of joints across foot, or of ankle-joint. Foot is contracted longitudinally, so as to measure nearly an inch shorter than left. Both inner and outer malleoli are in their proper places in relation to foot, but bones of leg lie in a line which falls behind the ankle-joint. In front there is a hollow, above what appears to me to be the ends of the tibia and fibula, fractured immediately above the ankle. In fact, I consider the case to be one exactly similar to that of which I have exhibited the dissected preparation, and his foot is as like the cast of Macnab's foot as it is possible for the foot of a different individual to be.

It is stated by Professor Cooper in his Dictionary, in both the articles "Trephine" and "Necrosis," that reproduction of bone in the cranium is rare, and that the deficiency of bone is *never* entirely obviated. The portion of the frontal bone which I now lay before the section exhibits a trepan-hole completely filled up with bone.

The man from whom I took this specimen was forty years of age when he died, in consequence of a severe injury received in a coal-pit. While attending him, I observed a crucial scar on his forehead, and felt under it a depression in the bone. He told me that when a lad of 12 or 14, having been engaged in one of those fights which delight the natives of the Sister Island, he sustained a fracture of the skull, and was trephined by a surgeon in Armagh, 26 or 28 years before. On his death, I removed the bone, and found the edge of the trepan-hole well-marked by a regular depression of above two-thirds of an inch, where the trephine had been applied; while below, the depression was irregular, probably from some splintering having taken place in that direction. The bone which fills up the hole is compact and translucent, except in lower part, where it is thickened, and projects a little internally. The mark is exactly in situation of left frontal eminence, which is destroyed by it, and just above the upper termination of the frontal sinus. This preparation of course settles the question of the complete filling up with bone in the affirmative.

---

TO THE EDITORS OF THE MEDICO-CHIRURGICAL REVIEW.

*Kilmarnock, 20th May*, 1840.

Gentlemen,

Never having observed any communications from the West of Scotland in your very widely circulated Journal, I have ventured to trouble you with a very brief sketch of a case, in the hope that it may be the means (if inserted) of stimulating some of my professional brethren, in this neighbourhood, to communicate some of those interesting facts and cases, which are not unfrequently presenting themselves to their notice. This, in my opinion, would not only invest the Medico-Chirurgical Review with a greater degree of interest to readers in this part of this country, but, if duly followed up, would lead to a more careful observation of facts, as well as extend to a wider sphere the benefit of many a long and extensive experience, which would otherwise be entirely lost. By giving the case which here subjoins, a corner in your excellent Journal, (if it seems worthy of that honour,) you will very greatly oblige,

Gentlemen,

Your most obedient servant,

JOHN THORNTON.

Mrs. M'A., æt. 31, the mother of four children, one of which she was still suckling, applied to me on the second day of April last, about what she considered a hernial tumour. She said she observed it first about eight years ago, and it was then no larger than a common garden bean. Since that time it had gone on gradually increasing in size. For the first four or five years it had not given her very great inconvenience, but now it had acquired such a size, as to have become extremely troublesome, preventing her in a great measure from walking or even from sitting with any degree of comfort. On examination, instead of hernia, I found it to be one of those large adipose tumours, which are not unfrequently found attached to various parts of the body. When handled it felt soft and elastic, but when more firmly pressed, a large hard and knotty body was felt in the interior. Its attachment extended from the symphysis pubis, downward along the whole labium pudendum on the left side as far as the anus. The tumour was quite moveable in all directions, and was evidently connected only with the cellular membrane underneath. When she stood upright it reached within four inches of the knee. It measured 12 inches round the base and eighteen round the apex. When out of bed she always kept it drawn up between the nates, and secured it there by a T bandage. On the 12th April, with the assistance of Dr. Paxton, I excised the tumour, which was contained in a capsule of condensed cellular membrane, and was completely dissected out without much difficulty. The hæmorrhage, which was chiefly venous, was considerable, in consequence of some large veins intersecting the tumour at its base. The urine for the first week was regularly drawn off by the catheter, and the wound, at the end of a month, was all but cicatrised, the patient expressing herself highly gratified at the removal of such a troublesome appendage. The growth, when laid open, appeared to consist entirely of adipose matter, and the hard body which was felt in its interior on firm pressure arose from a condensation of its internal structure.

The necessity for surgeons being minute in every instance with their examinations, is amply attested by the present case; no fewer than three different practitioners having been applied to, each of whom in their turn pronounced it to be hernia. This, in a great measure, may be accounted for, from the backwardness of the patient in allowing a scrutiny of a sufficiently precise nature to be instituted, as well as from the situation of the tumour itself. Had the true nature of the excrescence been properly ascertained at an earlier period of its history, she might have been saved from several years of great and distressing annoyance, as well as much of that pain which she must have endured during an operation for a large instead of a small growth.

A short time ago I assisted in the removal of a tumour of the same adipose structure, from the body of a female. Its base extended from the top of the dorsal vertebræ, over the top of the right shoulder, reaching almost as far round as the sternum. It had been four years in reaching this size, but during the last six months it had made such rapid progress as to have alarmed the patient. Had this case been neglected as the former one, it is impossible to say to what an extent it might have gone, and the difficulties that would have presented themselves in the removal of a tumour with such a wide-spread connexion, would of course have become greatly more formidable. A tumour of this description is generally unattended with pain, so that the patient is seldom led to make application about it until it has either begun to create annoyance from its bulk, or to create suspicion from the rapidity of its growth. This certainly is the reason, why so many excrescences are allowed to reach the extraordinary size which many of them are reported to have attained, before being subjected to the salutary influence of the knife. But in all cases where early advice is applied for, the patient ought at once to be made aware of the danger of procrastination, as well as of the safety and necessity of a timely operation. Adipose tumours growing from the labia pudenda seem by no means to be of unfrequent occurrence, and on that occount they merit the attention more particularly of provincial surgeons. In the eighteenth volume of the Medico-Chirurgical Review two cases are mentioned of a similar nature to the one noticed above. The one was suspended from the nates of a lady, but was found also intimately connected with the labium of the same side. It was removed by Mr. Lawrence, and is said to have been twice the size of that gentleman's head, which is reported to be a good one. The other was also attached to the left labium of a female, and was extirpated by Mr. Earle.

### BLOODLETTING IN HYDROCEPHALUS.

*(To the Editors of the Medico-Chirurgical Review.)*

GENTLEMEN,—The notice taken of Dr. Davis' work, in the recent number of your able periodical, shows that there is little novelty in either the pathology, or the therapeutics, of the experienced author. The inflammatory nature of acute hydrocephalus, and the necessity for copious depletion in its early stage, were first clearly demonstrated by the late eminent Dr. Rush, of Philadelphia; and the late Mr. Maxwell, who practised nearly forty years in this district, entertaining similar views, bled even more freely, I believe, than Dr. Rush. But that Dr. Maxwell *cured*, or to speak more philosophically (for the word cured is only fit for the advertisement of the charlatan,) that sixty out of ninety cases of hydrocephalus *recovered* under his care, is, I fear, saying too much, and may lead in future, as it has already done in many instances, to irreparable mischief. Dr. Maxwell was what is usually called a bold and decided practitioner—enjoyed great local celebrity,—possessed for a long series of years the unbounded confidence of the public, and was both sanguine and sanguinary in almost every acute disease that came under his notice. He bled freely not only in hydrocephalus, but in every complaint that in any way simulated or approached that dangerous disease.

During the last seven or eight years the lancet has been much seldomer used here, and blood under every circumstance more sparingly and cautiously abstracted. The *recoveries*, I have no hesitation in saying, have been quite as numerous, in proportion to the deaths, as formerly, and more rapid and satisfactory.

It must not be supposed, however, that I object to timely and full bleeding in well-marked cases of inflammatory hydrocephalus; on the contrary, I hold it of the first importance, that blood should be taken early in such cases, and from the jugular vein however young the patient may be.* I am only anxious to caution against returning to the frequent and profuse bleedings of former years.

We all know that there is a fashion in the affairs of medicine, which, like Byron's tide in the affairs of women, "when taken at the full leads God knows where." At the time I settled in Dumfries,—twenty-five years ago,—and for several years afterwards, I was just as sanguine and sanguinary as others, and so enamoured was I with the free way in which the vital fluid was spilt, and the apparent advantage of the practice in almost all cases among children simulating hydrocephalus, that in the summer of 1818 I transmitted you a short paper on the subject, which you did me the favour to publish in the October number of your Journal for that year.

The writings of Hall and Wardrop, on bloodletting, have done much good, and will, I trust, continue to have a salutary influence on the profession.

I am, Gentlemen,

Your very humble Servant

ARCHD. BLACKLOCK,

Late Surgeon R.N.

Dumfries, Oct. 20, 1840.

---

* In all acute diseases of infants and children when it is thought advisable to make a speedy impression by bleeding, the jugular vein, I think, ought to be preferred: the operation can be easily and safely performed by any person of ordinary adroitness; and the flow of blood may at all times be readily and permanently arrested by passing a fine cambric needle through the edges of the cutaneous orifice, and forming the twisted suture upon it with a common thread. Dr. Maxwell uniformly advised this method of securing the orifice, and often did it himself; for he was not one of those who hesitate to put a hand to when necessity requires.

CASES OF A FATAL WOUND OF THE INTESTINE INFLICTED BY A CLASP-KNIFE OPENING BY GYRATION: OF MASKED AND RAPID PLEURITIS AND PERICARDITIS; AND ALSO TWO REMARKABLE CASES OF JAUNDICE FROM OCCLUSION OF THE DUCTS, WITH DISTENTION OF THE GALL-BLADDER BY A COLOURLESS FLUID DESTITUTE OF BILE: AND OF ULCERATION OF THE RIGHT AND TOTAL DISAPPEARANCE OF THE LEFT KIDNEY: intended to illustrate the conservative efforts of Nature to avert danger, by means of compensating organs and vicarious functions. By Sir *David J. H. Dickson*, M.D. F.R.S.E. F.L.S. &c. Inspector of Hospitals.

On the afternoon of the 22nd April, 1840, a boat from H.M.S. Ætna, after landing ten patients at this hospital, proceeded to the opposite side of the Creek, with three army invalids, also from the North Coast of Spain, for the Military Hospital. While the officer went up with these men, the boat's crew (among whom were George Davis, æt. 21, and William Walker, æt. 20, messmates) amused themselves on the beach "skylarking," as it is termed, wrestling, &c. The latter had been thus engaged with another man, when the former came up behind, and after smearing his face with some dirt, ran off. Walker pursued him, whirling a closed knife attached to a long cord, which he held in his hand: but which, opening by the rapid gyration, (as it afterwards did, when the experiment was tried at the coroner's inquest) the point of the knife struck Davis, and wounded him in the left side of the abdomen, about half-way between the groin and umbilicus. When brought to the hospital, about 5 p.m. the wound, which was in a slanting direction, did not appear to be deep, nor to have penetrated the peritoneum: but, notwithstanding depletory measures, symptoms of intense inflammation supervened, and proceeded with such rapidity, that he died next evening at half past eight o'clock. What adds to the melancholy interest of this catastrophe, is, that although the result of the inquest was "accidental death," and no blame was imputed to Walker, who was considered of a mild, quiet, inoffensive disposition, and rather "soft," than otherwise, yet the death of his messmate so preyed upon the mind of the poor lad (who was a native of Dundee, and naturally rather low spirited) that he fancied he was an object of dislike to the officers and men; that he could not do anything to please them, &c. and, ultimately, he was sent to the hospital on the 4th of June, in a state of profound melancholia; having twice on that day attempted to commit suicide—in the first instance by strangulation, and afterwards by jumping overboard. By kindness and proper medical treatment, he recovered from this state of mental depression, and was discharged to the Cambridge, for which vessel he had expressed a preference; and I learned a few days afterwards that he was doing his duty like the other men, but I have not heard anything of him subsequently. The above particulars were related to me by Richard Wheeler (who was coxswain of the boat and afterwards a patient in this hospital), and others who witnessed the unfortunate accident: and I shall now only add the report of the dissection of Davis, made by Mr. Weale, my senior assistant, and which was laid before the coroner at the inquest.

*Sectio Cadaveris*, 20 hours, p.m.—The body was discoloured by livid blotches. The belly was tympanitic, and blood flowed from the mouth. On the left side of the abdomen there was a cut (evidently made by a sharp instrument) nearly three-quarters of an inch in length, and situated about the middle of a line drawn from Poupart's ligament to the umbilicus, though somewhat external to the latter. A probe could be introduced rather more than an inch into this wound, which ran in an oblique direction downwards and inwards, perforating the centre of the rectus abdominis muscle, and entering the cavity of the abdomen. From this opening some gas was observed to be gradually issuing; but its obliquity, and consequent valvularity prevented any other fluids from escaping. The epigastric artery lay close to the upper extremity of the wound, and a large branch of that vessel bounded it inferiorly, but both had escaped. The parietal, as well as the intestinal peritoneum was of a mottled, deep red colour, and the convolutions of the intestines were agglutinated by coagulable lymph; while there was a considerable quantity of serum, mixed with fæcal matter and gas, in the cavity of the abdomen, which had escaped by an opening in the jejunum, evidently in the direction of the wound: and, then capable of admitting a goose quill. On another knuckle of the bowel there was a deep red spot, probably from having been grazed by the point of the knife. The mucous membrane of the intestines and stomach was not inflamed, but the latter contained some feculent looking fluid. The lungs were much congested; the bronchial lining membrane was of a deep dusky red colour, and the trachea contained semi-coagulated blood.

It is too probable that no treatment would have availed in the present case: and I need not here dwell upon the vast importance of the *earliness* of energetic measures in such instances; in consequence of the incomparably greater rapidity and danger of inflammations of the serous than of the mucous membranes; or of those of the abdomen than of the thorax: but as illustrating the occasionally obscure and masked, yet rapid and dangerous character of those of the chest also, I may here adduce the autopsy of two late cases of *pleuritis* and *pericarditis*: in the one who was admitted for "fever," on the 10th, and died on the 18th of July, but in reality with empyema of the left pleura, the effusion, though intolerably offensive and exceeding a gallon in quantity so as to displace the heart to the right side, had speedily taken place; and the compressed lung and all the pleural reflexions were covered with false membrane; large shreds of which also hung from the costal lining, which was gangrenous in several places.

In the other instance, the patient, an officer, had been ill for about a week only, but neglected at first to apply for assistance, and had been so far relieved by depletion as not to be considered in any danger, until the morning when he was admitted into this hospital, moribund; yet in so short a period, the heart and pericardium (which contained at least 24 ounces of puriform fluid, with masses of natant lymph in it) had become so thickly coated with false membrane as to present a reticulated appearance, while the serous membrane beneath was of a deep red colour, from vascular injection.

I shall not here detail the well known processes by which Nature, by the absorption of old and the deposition of new matter, effects the reparation of injuries inflicted by accident or disease in the various structures of the body,—as in the case of fractures—the incarnation of wounds—the prevention or arrest of hæmorrhage, &c. or the wonderful efforts of this conservative power to repair, or limit the mischief which may have been produced by the effusion, and re-absorption of pleuritic and other accumulations of fluid,—the conversion of false membrane—the sprouting of new to compensate for the loss of old vessels—the occasional lining and approximation of tubercular and other cavities—the closing of apoplectic cysts, &c. &c. but will conclude by adducing only two very recent instances, illustrative of the attempts of this ever-busy power—whether partially successful or abortive—to arouse compensating organs to increased action, and thus, by means of vicarious functions or vicarious discharges, as by the exhalants &c., to supply the office, or make up for the deficiencies of such as are impeded or suppressed, in the latter point of view. In the following case in which the occlusion of the duct completely prevented the admission of bile into the gall-bladder, the secretion even to repletion of a colourless ropy fluid like saliva, from its internal surface, and the effusion of blood, and apparently of bile with which the system was loaded, by the intestinal exhalants, are singularly curious and interesting.

*John Holmes,* S. æt. 52, was admitted with what is vulgarly termed the "black jaundice," on the 14th, and died on the 22nd of July last. He stated that he had never been discoloured before; that he imputed it to having received a blow on the back; and that he had been ill only three weeks, which is incredible, considering the extent of disease afterwards detected. The face and extremities were of a dark mahogany colour; while the body exhibited the extraordinary appearance of purple spots, on a deep yellow ground. The liver was tender, and much indurated; and not only was the urine of the deepest hue, but the ejections from the stomach and bowels, instead of being pale, consisted of a very dark, bloody, offensive matter, which appeared to be a mixture of mucus, blood, and bile, thrown out by the intestinal exhalants: for from the occlusion of the gall ducts, the system was so thoroughly imbued with this fluid, that the white tissues, and even the lining membrane of the arteries were deeply dyed with bile. I need scarcely add that where the portal and hepatic circulation is much obstructed, by what is termed " scirrhus," or other structural disease of this viscus, or of the heart, &c. congestions take place in other organs, which nature attempts to relieve by vicarious discharges. Hence, as in a late instance, we occasionally meet with melæna, or other profuse evacuations, *supra infraque,* where no evidence of disease of the mucous surface can be detected—and, more frequently, with serous, or sanguineous effusions, in the shape of dropsy, or hæmorrhage from the stomach, bowels, lungs, nostrils, &c. The extent of such obstruction is strongly portrayed in the following dissection of *Holmes,* ten hours p.m.

The abdomen contained a small quantity of yellow serum. The liver was thickly studded with large tubera, especially its concave surface, though it was tuberated throughout;—but its colour was lighter, and its situation better defined than in health;—the paler tissue forming a yellowish olive ground, which rendered the vascular parts more distinct. On cutting it, no blood escaped, but a clear, oily-looking fluid of the consistence of synovia, flowed in considerable quantity from the incised surfaces.

The gall bladder was enormously distended by a perfectly colourless, but somewhat ropy fluid, like saliva; but destitute of any appearance of bile whatever; and the cystic and hepatic ducts were completely obliterated, and lost, in a scirrhous, pancreatic-looking mass, deposited between the layers of the lesser omentum; and involving the great end of the pancreas, which did not seem to be further diseased:—its duct being pervious to within an inch of its extremity. The hepatic vessels, vena cava, aorta, and the upper third of the duodenum were inseparably accreted together by this morbid deposit, which surrounded the aorta as far as the superior mesenteric artery: and must have caused considerable obstruction of the inferior vena cava.

The portal system was unusually bloodless. The stomach and upper part of the intestines contained a great quantity of a dark offensive fluid, resembling a mixture of bilo-grumous blood and matter; and the mucous surface had an ecchymosed appearance.

The spleen was healthy, the kidneys were large;—internally of a yellowish-olive colour; and a bilious looking fluid exuded so freely on section, that they seemed as if they had been soaked in bile; and, as already noticed, all the white tissues, and even the lining membrane of the arteries, were deeply tinged with this fluid.

The last which I shall mention was, also, a very extraordinary case of ulceration of the right, and the absence, or total disappearance of the parenchyma of the left kidney; which, whether resulting from original vice of conformation, or, which is more probable, absorption from disease, is rendered still more remarkable by the statement of the surgeon that the man, a quarter-master, from the Vanguard, at Portsmouth, had been actively employed in raising volunteers at Devonport, and had been taken ill only three days previously to his admission into the hospital.

*Isaac Abrahams.* æt. 43, was received with symptoms of gastro-enteric, and renal disease, on the 14th, and after laying some days in a semi-comatose, or delirious state, latterly with suppression of urine, and copious perspirations of a strongly urinous, ammoniacal smell, died on the 23d July, 1840.

*Sectio cadaveris,* 35 hours p.m. The vessels on the surface of the cerebrum were tinged with blood; and the arachnoid membrane was raised by a copious effusion of serum, emitting an odour like new-made hay. The substance of the brain was firm, and presented, on section, a quick succession of numerous bloody points; but no fluid was found in the ventricles at its base, nor in the spinal canal.

The contents of the thorax, as in the preceding case, and also of the abdomen, appeared to be nearly normal; with the exception of a large, oblong, fluctuating tumor, which at once attracted attention, in the left hypochondrium. It projected beyond the colon, which it pushed backwards and towards the side. The supra-renal capsule was attached to its superior extremity. The renal vessels entered it about one-third lower;—and about two inches and a half below this, the ureter issued between its coats obliquely upwards, and then turned downward in the usual direction, and of the natural size. Upon being removed this tumor measured about sixteen inches round, and twenty-three inches in its oblong circumference. It was covered partially by the peritoneum, and entirely by the usual fibrous coat; between which, and the internal mucous surface, a considerable number of large blood-vessels ramified. It was completely distended by a perfectly limpid, inodorous fluid, like water, which, on puncturing it, escaped.

The parenchymatous, and indeed the whole structure of the kidney, had entirely disappeared; though some faint traces of the papillæ were still visible. The mouth of the ureter was perfectly distinct, and the opening permitted the introduction of a probe for more than an inch, while the obliquity of its passage through the coats, impeded its further progress,—and acting as a valve, for the same reason, prevented the fluid collected in this large sac from descending into the bladder; for, otherwise, the canal seemed to be permeable throughout. The right kidney, having a compensative or double duty to perform, was enlarged, and the infundibula and the commencment of the ureter for some inches were dilated; while the coats of the latter were also thickened, so that it measured about the size of the thumb in circumference. The substance of the kidney had evidently suffered from recent inflammation, and was thickly studded with bloody points. Its pelvis, which was distended with brownish turbid urine, was covered with ash-coloured false membrane; which was partially detached, and exposed a raw and ulcerated surface beneath, extending into the ureter; which, as already noticed, was dilated, but became gradually narrower, until it exhibited the usual size and appearance, within five inches of the bladder. The latter organ was much contracted in size, but apparently in a healthy condition.

DAVID J. H. DICKSON.

Royal Naval Hospital, Plymouth,
1st September, 1840,

